# Lumbar Sympathetic Block with Botulinum Toxin Type A and Type B for the Complex Regional Pain Syndrome

**DOI:** 10.3390/toxins10040164

**Published:** 2018-04-19

**Authors:** Yongki Lee, Chul Joong Lee, Eunjoo Choi, Pyung Bok Lee, Ho-Jin Lee, Francis Sahngun Nahm

**Affiliations:** 1 Department of Anesthesiology and Pain Medicine, Seoul National University Bundang Hospital, Seongnam 13620, Korea; yongkilee.md@gmail.com (Y.L.); ejchoi@snubh.org (E.C.); painfree@snubh.org (P.B.L.); zenerdiode03@gmail.com (H.-J.L.); 2 Zeropain Clinic, Seoul 02830, Korea; may97lee@yahoo.com

**Keywords:** botulinum toxin, complex regional pain syndrome, lumbar sympathetic ganglion block, pain

## Abstract

A lumbar sympathetic ganglion block (LSB) is a therapeutic method for complex regional pain syndrome (CRPS) affecting the lower limbs. Recently, LSB with botulinum toxin type A and B was introduced as a novel method to achieve longer duration of analgesia. In this study, we compared the botulinum toxin type A (BTA) with botulinum toxin type B (BTB) in performing LSB on patients with CRPS. LSB was performed with either BTA or BTB on patients with CRPS in their lower extremities. The length of time taken for patients to return to the pre-LSB pain score and the adverse effect of LSB with BTA/BTB were investigated. The median length of time taken for the patients to return to the pre-LSB pain score was 15 days for the BTA group and 69 days for the BTB group (*P* = 0.002). Scores on a visual analogue scale decreased in the patients of both groups, and no significant adverse effects were experienced. In conclusion, the administration of either BTA or BTB for LSB is a safe method to prolong the sympathetic blocking effect in patients with CRPS. BTB is more effective than BTA to prolong the sympathetic blocking effect in CRPS patients.

## 1. Introduction

Complex regional pain syndrome (CRPS) is a rare chronic pain syndrome that causes sensory symptoms such as spontaneous pain and allodynia, as well as motor and autonomic nervous system symptoms [1]. It is an intractable pain disorder that requires a multimodal treatment approach, and there is currently no single treatment method that is specific to CRPS [2]. Of the existing methods, sympathetic blocks are known to reduce pain as well as improve motor and autonomic nervous system functions [3]. Despite the small body of research on the effectiveness of sympathetic blocks, the procedure is widely used for the treatment of CRPS [3,4].

A nerve block using local anesthetics is generally administered to the lumbar sympathetic chain in patients with CRPS in their lower extremities. The blocking effect is temporary in most patients, and therefore, achieving a more long-term effect necessitates one or more of the following additional procedures: repeated sympathetic blocks with local anesthetics, radiofrequency thermocoagulation or neurodestructive procedures that use alcohol or phenol. The merits of a chemical neurolysis of the sympathetic nerve using alcohol or phenol include that it is a simple, economical procedure that can elicit a relatively long-term treatment effect. However, complications can occur, such as genitofemoral neuralgia, which has a prevalence of about 4–10% [5,6]. Moreover, post-sympathectomy neuralgia can occur after chemical sympathetic neurolysis with alcohol or phenol [7]. Radiofrequency thermocoagulation is reported to have similar long-term effects to those of neurodestructive procedures that use phenol [6]. Due to the anatomical location of the lumbar sympathetic chain, however, difficulties have been associated with the procedural method in terms of needle positioning during radiofrequency lesioning [8]. Additionally, radiofrequency lumbar sympatholysis is related to post-sympathectomy neuralgia [9], similar to the chemical sympatholysis.

Botulinum toxin (BT) inhibits the release of acetylcholine from cholinergic nerve endings and is typically used in the treatment of dystonia [10]. The sympathetic blocking effect can be obtained by injecting BT at the sympathetic ganglion, because presynaptic fibers of sympathetic ganglia are also cholinergic. There have been several reports on the prolonged sympathetic blocking effect of BT. When botulinum toxin type A (BTA) was injected into the superior cervical ganglion of a rabbit, the sympathetic blocking effect lasted for a minimum of 1 month and no pathological changes occurred [11]. Another study reported on BT use for a ganglion impar block in patients with chronic perineal pain; a ganglion impar block that used BTA was performed and resulted in pain relief for 3 months and longer [12]. Moreover, Carroll et al. [13] reported that a sympathetic block that used BTA in patients with CRPS in their lower extremities yielded a blocking effect (without any serious complications) for approximately 2 months longer than a block that only used local anesthetics. 

Differences exist between BT type A and type B (BTB) with regard to their mechanism of action and target proteins, and moreover BTB can block the release of acetylcholine in the cholinergic nerve fiber [10,14]. Based on this mechanism of action, BTB has been used in clinical practice for treating cervical dystonia [15], adductor muscle spasms [16], piriformis syndrome [17], subacromial bursitis [18], overactive bladder [19] and hyperhidrosis [20]. However, only a few studies have compared the effects of BTA and BTB. When BT was used in patients with cervical dystonia, type A had a longer duration of action than type B [21], but there is no clear conclusion regarding the clinical effect of this difference. Additionally, BTB has been reported to spread into the surrounding tissue less than BTA [14]. With these background studies in mind, we searched the literature for the use of BTB for a lumbar sympathetic block (LSB), but only one case report was found [22]. Furthermore, there has been no published research comparing the effect of the two types of BT when used for LSB. Therefore, the purpose of this retrospective observational study was to compare the effect of BTA and BTB when performing LSB in patients with CRPS in the lower extremities.

## 2. Results

A total of 18 patients were included in this study. The demographic data of the patients are shown in Table 1. 

One patient in group A and one patient in group B complained of dizziness after the procedure, but the symptoms improved within a week without any particular treatment.

**Figure 1:**
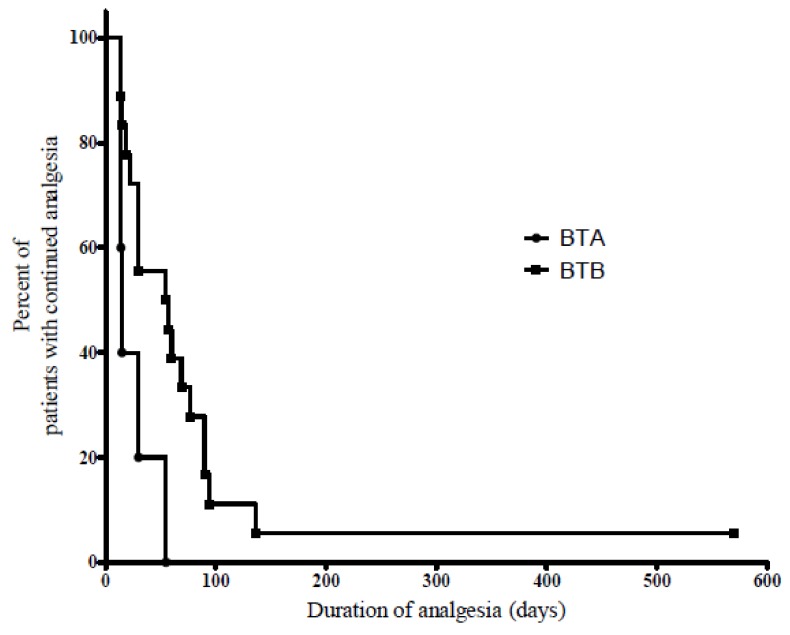
Log-rank analysis of the duration of analgesia. The median duration of analgesia was 15 (95% CI, 12.9–17.1) days for the BTA group, and 69 (95% CI, 45.5–92.5) days for the BTB group. There was a significant difference in the duration of analgesia between the two groups (*P* = 0.002).

There were no significant differences among the demographic characteristics between the groups. The median duration of observation was 56 days (range 14–570 days). The time taken to return to the pre-procedure VAS score was significantly longer in group B than group A (Figure 1). The median time to return to baseline pain was 69 days in group B (95% CI, 45.5–92.5 days) compared to 15 days for group A (95% CI, 12.9–17.2 days) (*P* = 0.002). The VAS scores that were recorded 1 week after the procedure were found to be significantly lower in both groups compared to those which were recorded prior to the procedure. The median VAS score change that occurred between these time points was 2.5 (range 1.0–6.0) (*P* = 0.043) in group A and 3.0 (range 0.5–5.0) (*P* = 0.001) in group B. However, there was no significant difference in the delta-VAS score before and after treatment in both groups 1 week after the procedure (*P* = 0.633).

## 3. Discussion

In our study, the sympathetic blocking effect of BTB was longer than that of BTA in the patients with CRPS in the lower limbs. Chemical sympathectomy is used widely: it is economical, easy to perform and anticipated long-term effects. In patients with neuropathic pain or CRPS, however, there is no high-quality evidence for the use of chemical sympathectomy, and it is only recommended for use with caution only in clinically selected patients [4,23]. When sympathetic nerves are destroyed through chemical, surgical, or radiofrequency methods, further neuropathic pain can occur due to nerve destruction [24]. Post-sympathectomy neuralgia is a syndrome of pain with a burning nature, which can last 2–3 months [25]. This syndrome can be more severe and debilitating than the initial pain complaint [24], which is thought to be a result of the sensitization of the viscerosomatic nociceptive spinal neuron caused by the sympathectomy. An additional contributing factor may be differentiation hyperactivity in the sensitized neurons as a result of the axotomy [4,26]. Due to the anatomical location, genitofemoral neuralgia can also occur after chemical lumbar sympathectomy [5,6]. Additionally, surgical sympathectomy has been found to produce postsympathectomy neuralgia in up to 39% of patients [27]. 

BT has seven serotypes, and among these, types A and B are used for the treatment of various conditions. The target site of the toxin is the neuromuscular junction, and the toxin works by dissolving the SNARE [soluble N-ethylmaleimide sensitive factor attachment protein (SNAP) receptor] complex which functions in exocytosis. Type A works on SNAP25, and type B works on synaptobrevin. As a result, BT blocks the release of acetylcholine and exocytosis, causing flaccid paralysis [10]. Therefore, BT is used in the treatment of pain that occurs from dystonia and excessive muscle contraction [28]. 

BT is also reported to be effective in neuropathic pain syndromes, such as trigeminal neuralgia, postherpetic neuralgia, and CRPS, as well as pain caused by dystonia [13,29,30,31]. The effectiveness of BT is due not only to the repression of the secretion of acetylcholine, but also to the blocking of the exocytosis of various pain-related neurotransmitters such as substance P and calcitonin gene-related peptide [32,33,34]. Therefore, there can be additional benefit in using BT for sympathetic block, not only prolonging the duration of sympathetic block but also interrupting the cascade of pain-related neurotransmitters in CRPS. 

Few studies have directly injected BT near the nerve, especially near the sympathetic ganglion [35]. In the first study to report on the action and effect of BT on the sympathetic ganglion, BTA was injected into the surgically exposed superior cervical ganglion of a rabbit [11]. The effect of BTA was compared through miosis to a control group that received normal saline, and there was a dose-dependent sympathetic blocking effect of BTA. However, there was no significant change in the number or shape of the nerve cells as compared to the control group. In rabbits in which miosis was observed, the mean time of duration was 5.3 weeks. These findings suggest that BT causes a sympathetic block without causing any histological changes, and this blocking effect reflects the largest difference when compared to chemical sympathectomy using alcohol or phenol. 

There was a crossover study that compared the duration of pain relief when using only local anesthetics versus using both local anesthetics and BTA together in performing a lumbar sympathetic ganglion block in nine patients with CRPS in the lower extremities [13]. When BTA was used, the median time taken to return to the level of pain prior to the procedure was 69 days, whereas only 8 days were required when only local anesthetics were used. In addition, with the exception of one patient who complained of nausea and vomiting, which improved without any particular treatment, there were no incidents of adverse effects. This shows that a long-term blocking effect can be safely attained by injecting BTA near the sympathetic ganglion in humans. 

A few studies have compared the duration of the blocking effect when using BTA or BTB in a sympathetic block. In a randomized double-blind study that compared the duration of the effect using BTA or BTB in patients with cervical dystonia, similar durations of 14.0 weeks and 12.1 weeks were found, respectively, and this small difference was statistically significant [21]. Similar results were observed in preclinical research [36]. In another study that compared the effect of BTA and BTB in toxin-naïve cervical dystonia patients, no significant difference in the duration of action was found, and the durations were 13.1 weeks and 13.7 weeks, respectively [37]. 

In our study, BTB resulted in a 4-fold longer duration compared to BTA. These results are contrary to preceding studies; however, the disease and the treatment areas are different. Previous researches that compared the two types of BT have been limited to cervical dystonia and aesthetic treatments. Both of these procedures can involve injecting BT directly into the muscle, and the results may differ when injecting BT closer to the sympathetic ganglia. When BT was injected into the paws of the monkeys, the spreading of toxin was smaller with BTB than with BTA [14]. This result implies that the BTB can be more focused than BTA at the targeted tissue. However, this result conflicts with clinical data that has reported more side effects, such as dry mouth and dysphagia, when BTB was used [38]. Nevertheless, these adverse effects were mostly light to moderate and tended to decrease when injections were repeated, which suggest that rather than occurring from the toxin spreading from the injected area, the side effects likely occurred because of the high sensitivity of BTB to cholinergic autonomic neurons, particularly postganglionic neurons, e.g., the M3 receptor [39]. The results of the present study, including longer duration of action in BTB can be explained in light of the fact that BTB spreads less to surrounding tissues than BTA and that BTB works more specifically on cholinergic autonomic neurons in the sympathetic ganglia.

Our result that LSB with BTA showed only 15 days (95% CI, 12.9–17.2 days) of median time to return of baseline pain was quite short compared to the previous study result. The previous study by Carroll et al. reported that the 95% confidence interval of the median duration of LSB with BTA was 12–253 days [13], which was very wide. Only three of the eight patients in the study of Carroll et al. showed significantly prolonged effect of BTA. Another possible reason is that the study design of the previous manuscript was ‘cross-over design’. According to the article by Nordmann et al. “the successive LSB provided increasing duration of completely pain free period” [40]. Therefore, the repetitive LSB with local bupivacaine only and bupivacaine with BTA in the study by Carroll et al. resulted in the prolonged effect of LSB.

There are limitations to our study. First, this was a retrospective study. Due to the lack of information on BTB use for LSB, there were difficulties in designing and performing a prospective double-blinded study. We used BTA 100 IU and BTB 5000 IU for LSB. In the previous studies which compared the BTA with BTB in the patients with cervical dystonia, the dose ratio of 1 U BTA was comparable to 40–67 U BTB [21,37]. From this, we set the equivalent dose ratio of 1 U BTA to 50 U BTB (BTA 100 = BTB 5000 U) in our study. We think our study can provide a foundation for future well-designed studies on BT use for LSB. Second, because of the retrospective design, we could not measure various changes in symptoms and signs (e.g., oedema, cold sensation and mechanical allodynia), but could measure only the VAS scores, in each patient. Third, the sample size was quite small; however, CRPS is a rare, intractable disorder; therefore, it was not easy to recruit many CRPS patients for a 3-year study period. A multi-center study is necessary to recruit many patients. 

## 4. Conclusions

In this study, the authors confirmed that both BTA and BTB can be used with no serious side effects when used for an LSB in patients with CRPS type 1 in their lower extremities. In terms of the duration of pain relief, BTB was found to be more effective than BTA. However, this research is limited in that it was a retrospective study and did not include many patients in the BTA group. A well-designed, randomized prospective study is needed to compare the effects of the two types of BT in LSB.

## 5. Materials and Methods

### 5.1. Study Participants

This retrospective observational study was approved by the institutional review board of our hospital (IRB No. B-1311/228-102), and written consent was obtained from the patients and their guardians after they received an explanation of the methods and the purpose of the procedure as well as recent research results. 

The inclusion criteria for patients consisted of the following: (1) patients who visited the pain center at our university hospital and were diagnosed with CRPS type 1 in the unilateral lower extremities from March 2010 to June 2013 and (2) patients who received LSB with either BTA or BTB. The diagnosis of CRPS was made according to the criteria known as the ‘Budapest research criteria’ [41], which is a revised version of the criteria that were originally proposed by the International Association for the Study of Pain (IASP) [42]. According to the criteria, the patients should meet at least one symptom in all four categories (sensory, vasomotor, sudomotor/edema, and motor/trophic change) and at least one sign in two or more categories. Furthermore, LSB with BT was performed on patients who had previously shown significant pain reduction (50% or more) after LSB with 5 mL of 0.25% ropivacaine. The exclusion criteria consist of the following: (1) patients who had received any neuro-destructive procedure on the lumbar sympathetic ganglia using radiofrequency thermocoagulation or chemicals (alcohol or phenol) or (2) patients who had received a sympathetic block within 6 months.

### 5.2. LSB Procedure

**Figure 2:**
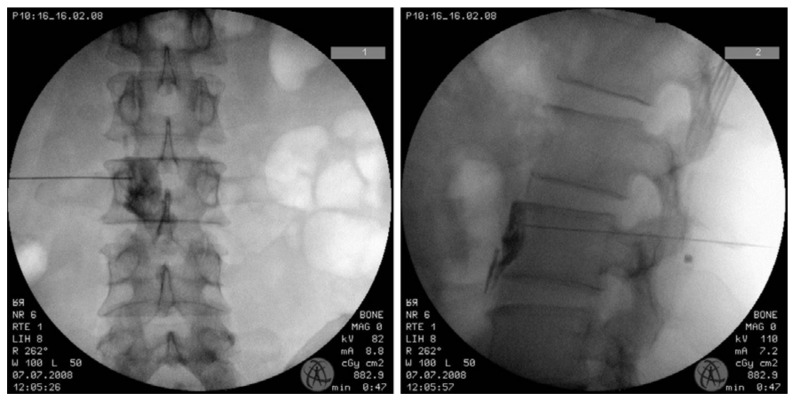
Spread of the contrast agent during the lumbar sympathetic block. The contrast agent was confined to the anterolateral border of the vertebral body without any psoas spread laterally or intravascularly. (**1**) Antero-posterior view. (**2**) lateral view of the fluoroscopy.

Percutaneous LSB was performed in the operating room with fluoroscopic guidance (Figure 2). The patient was placed in a prone position on a radiographic table with a 15-cm-high pillow underneath the patient’s abdomen to reduce lumbar lordosis. The patient was given an intravenous infusion of lactated Ringer’s solution and was monitored by pulse oximetry, electrocardiogram and blood pressure readings during each procedure. For the LSB procedure, a 21-gauge 15 cm Chiba needle (Cook Inc., Bloomington, IN, USA) was advanced under the oblique projection of a C-arm fluoroscopy. When the Chiba needle was inserted properly at the anterolateral side of the L3 vertebral body, 1–2 mL of contrast agent (Omnipaque^®^, Nycomed Ireland Ltd., Cork, Ireland) was injected to confirm adequate spread around the target site (anterolateral border of the vertebral body) and to identify any intramuscular or intravascular spreading. When the contrast agent did not spread adequately, the same procedure was repeated by inserting another needle at the L2 vertebral level. In group A, BTA 100 IU (Botox^®^, Allergan Inc., Irvine, CA, USA) combined with 0.25% ropivacaine (2.5 cc) was injected; in group B, 5000 IU BTB (Myobloc^™^, Solstice Neurosciences Inc., Louisville, KY, USA) combined with 0.25% ropivacaine (2.5 cc) was injected. When the needle was inserted only at the L3 level, the entire dosage of the medication was injected. Furthermore, when the needle was inserted at the L2 and L3 level, the dosage of BTA or BTB was divided in half and combined with 0.25% ropivacaine (2.5 cc) for injection at each level. After LSB, Skin temperature was measured 30 min after the LSB. The sympathetic block was considered successful when the skin temperature of the affected side increased by ≥2 °C.

### 5.3. Data Collection and Statistical Analyses

From the patients’ medical records and demographic data, the VAS scores prior to and 1 week after the procedure were examined, and the number of elapsed days until the VAS score returned to the pre-LSB state was recorded. The occurrence of any adverse effects or complications was also examined. The primary endpoint—the time taken to return to the baseline VAS score—was analyzed using Kaplan–Meier analysis. The difference between the two groups was analyzed by the log-rank test. The secondary endpoint—the VAS score change from before to after the procedure—was expressed as the median with a range and was tested using the Wilcoxon signed rank test. PASW^®^ software version 18.0 (Chicago, IL, USA, 2009) was used for the statistical analysis, and *P*-values < 0.05 were considered statistically significant.

## Figures and Tables

**Table 1:** Demographic data.

Variables	BTA Group (*n* = 5)	BTB Group (*n* = 13)
Gender (M/F)	5/0	10/3
Age (median, range) years	26 (21–43)	23 (20–47) M 23 (20–39)/F 36 (23–47)
10-cm VAS score (median, range)		
Pre-LSB	7.5 (3.5–8.5)	6.0 (2.0–10.0) M 5.5 (3.5–10.0)/F 6.0 (2.0–10.0)
Post-LSB	3.0 (2.0–5.0)	3.0 (1.0–7.0) M 3.0 (2.0–6.0)/F 5.0 (1.0–7.0)

Values represent the number of patients. M: male, F: female, BTA: botulinum toxin A, BTB: botulinum toxin B, VAS: visual analogue scale, LSB: lumbar sympathetic block.
